# Burden of congenital and hereditary anomalies in the population of Azad Jammu and Kashmir, Pakistan

**DOI:** 10.12669/pjms.40.11.8687

**Published:** 2024-12

**Authors:** Filza Khursheed, Rubbiya Farid, Saba Rafique Qureshi, Sajid Malik

**Affiliations:** 1 Filza Khursheed, M.Phil, Human Genetics Program, Department of Zoology, Faculty of Biological Sciences, Quaid-i-Azam University, 45320 Islamabad, Pakistan; 2 Rubbiya Farid, M.Phil, Human Genetics Program, Department of Zoology, Faculty of Biological Sciences, Quaid-i-Azam University, 45320 Islamabad, Pakistan; 3 Saba Rafique Qureshi, M.Phil, Human Genetics Program, Department of Zoology, Faculty of Biological Sciences, Quaid-i-Azam University, 45320 Islamabad, Pakistan; 4 Sajid Malik, PhD, Human Genetics Program, Department of Zoology, Faculty of Biological Sciences, Quaid-i-Azam University, 45320 Islamabad, Pakistan

**Keywords:** Birth defects, Neurological disorders, Limb defects, Descriptive epidemiology, Consanguinity

## Abstract

**Objectives:**

This study was aimed to elucidate the prevalence-pattern of congenital and hereditary anomalies (CA) in the population of Azad Jammu and Kashmir (AJK), which is a logistically difficult area in the north-east of Pakistan and in the foot-hills of Himalayas.

**Method:**

A cross-sectional clinico-epidemiological study was carried out during 2018-2020 in three districts of AJK and individuals/families with CA were recruited from hospitals, public places and through door-to-door surveys. The anomalies were classified with the help of specialized clinicians and their definitions were sought from OMIM and ICD-10 databases. Descriptive statistics was employed.

**Results:**

A total of 1193 independent individuals/families with certain type of CA were included. The CA were categorized into 10 major and 85 minor entities. Among the major categories, neurological disorders had the highest representation (n=403; proportion: 0.338; 95%-CI: 0.311-0.365), followed by limb defects (n=362; prop.: 0.303; 95%-CI: 0.277-0.330), sensorineural defects (n=187; prop.: 0.157; 95%-CI: 0.136-0.177), musculoskeletal disorders (n=64), visual impairments (n=64), ectodermal disorders (n=40), orofacial disorder (n=34), blood disorder (n=11), metabolic disorders (n=8), and others (n=20). The anomalies with sporadic presentations were twice as common as familial cases, and there was remarkably high preponderance of isolated cases compared to syndromic presentations (82% vs. 18%, respectively). The parental consanguinity was observed in 63% cases and was statistically significantly higher in familial cases compared to sporadic.

**Conclusions:**

Majority of the anomalies observed in this cohort are of severe nature rendering high morbidity burden on the population and require early detection, intervention and management.

## INTRODUCTION

The developmental disorders of fetus and embryo are acknowledged as birth defects or congenital anomalies (CA). These defects include anomalies in structure, function, and metabolism, and they may be fatal at times or lead to disability that can be physical as well as mental.^1^ Depending upon the phenotypic and clinical criteria, CA can be defined as major or minor anomalies. Major malformations generally cause functional impairments. On the other hand, minor anomalies are those that do not result in functional impairments and may not require medical interventions.^2^ The global birth prevalence of CA is 2-3%, with wide regional variation. The prevalence varies from 6/1000 in Barbados to 28/1000 in the UK.^3^,^4^ CA have been reported to be a significant cause of mortality and morbidity in the developed as well as developing countries.^3^,^5^

Due to various reasons, the prevalence of CA is very high in Pakistan compared to the developed countries. These reasons include a high incidence of consanguinity, marriages at young ages and extended sibships, inadequate antenatal care, high-risk pregnancies and poor management.^6^,^7^ In the rural areas of Pakistan, the majority of the childbirths take place at home and the CA emerging in these populations remain underrepresented in the hospital-based cohorts. Epidemiological studies may be challenging to conduct in the rural and remote populations due to lack of skilled enumerators and resource persons, and meager resources.^8^,^9^ This study reports the prevalence-pattern of CA in the Azad Jammu and Kashmir (AJK), which is a remote population in mountainous terrain at the North-East of Pakistan.

## METHODS

This descriptive clinico-epidemiological study was carried out during 2018-2020 in AJK. The population comprises 4.5 million individuals, the majority of which reside in rural areas with urban-to-rural ratio of 12:88.^10^ There is a high trend of consanguineous marriages in Kashmir.^11^ The health system is inadequate and is not able to support masses residing in rural areas. An estimated 62/1,000 born children die in the first year of their lives and there is one bed for 1310 individuals in the hospitals. The region is underserved in basic infrastructure, drinking water and sanitation.

### Ethical Approval:

The study was approved by the Ethical Review Committee of the Quaid-i-Azam University (Ref# DAS/19; July 3, 2019).

### Methodology and Data collection:

All data were acquired according to the Helsinki-II declaration.^12^ The data on individuals/families having a certain type of CA were acquired from OPDs of district hospitals like Ambor Hospital Muzaffarabad, Combined Military Hospital (CMH) Poonch and CMH-Muzaffarabad, and DHQ-Bhimber. In small towns, the individuals were ascertained from rural health units and through door-to-door surveys. The fieldwork was carried out with the help of local resource persons, midwives and lady-health-visitors. Each index individual with CA was physically examined with the help of local physicians and specialized doctors. Cases with traumatic and acquired anomalies were not included.

### Classification of anomalies and statistical analyses:

The definition of each anomaly was searched through online databases like OMIM (http://www.omim.org/) and ICD-10 (https://icd.who.int/browse10/2019/en). Syndromic cases were identified with respect to the more severe symptoms in an organ-system in the following order: neurological disorders, musculoskeletal defects, visual impairments, sensorineural anomalies, and limb defects. Only the index individual in a particular household was enumerated. Descriptive summaries were generated and distribution of index cases was established across demographic variables. The odd ratios (OR) of index males in contrast to index females were calculated and the lowest risk category in each variable was taken as reference. For each CA, the proportions and 95% confidence intervals (95% CI) were estimated from the total number of anomalies.

## RESULTS

### Sample characteristics:

A total of 1193 independent individuals/families with certain types of CA were recruited. Male and female index individual were 691 and 502, respectively. The demographic attributes of the index cases are presented in Table-I.

**Table-I:** Demographic attributes of index cases and odds of affected males.

Variable	Male, %	Female, %	Total	Proportion	95% CI	OR^#^
***District***						
Muzaffarabad	401 (58)	293 (59)	694	0.582	0.554-0.610	Ref.
Poonch	142 (21)	97 (19)	239	0.200	0.178-0.223	0.97
Bhimber	148 (21)	112 (22)	260	0.218	0.195-0.241	1.03
Total	691 (100)	502 (100)	1193	1.000	-	
Origin*						
Rural	455 (66)	363 (72)	818	0.686	0.659-0.712	1.35
Urban	236 (34)	139 (28)	375	0.314	0.288-0.341	Ref.
Age ranges (years)*						
Up-to 9	230 (33)	169 (34)	393	0.334	0.308-0.361	Ref.
>9-19	259 (37)	141 (28)	400	0.335	0.308-0.362	0.74
>19	202 (29)	192 (38)	394	0.330	0.304-0.357	1.29
***Language***						
Hindko	372 (54)	280 (56)	652	0.547	0.518-0.575	Ref.
Pahari	166 (24)	124 (25)	290	0.243	0.219-0.267	0.99
Punjabi	121 (18)	81 (16)	202	0.169	0.148-0.191	0.89
Others	32 (5)	17 (3)	49	0.041	0.030-0.052	0.71
***Caste-system***						
Rajpoot	90 (13)	79 (16)	169	0.142	0.122-0.161	1.80
Chaudhary	86 (12)	73 (15)	159	0.133	0.114-0.153	1.74
Sadozai	88 (13)	70 (14)	158	0.132	0.113-0.152	1.63
Awan	71 (10)	59 (12)	130	0.109	0.091-0.127	1.70
Mughal	67 (10)	36 (7)	103	0.086	0.070-0.102	1.10
Abbasi	41 (6)	27 (5)	68	0.057	0.044-0.070	1.35
Syed	45 (7)	22 (4)	67	0.056	0.043-0.069	Ref.
Others	203 (29)	136 (27)	339	0.284	0.259-0.310	1.37

* Differences in the distribution were statistically significant #Odd ratio of males compared to females.

### Categorization of anomalies:

The CA were characterized into 10 major and at least 85 minor entities (Table-II). Among the major groups, neurological disorders had the highest representation (n=403; proportion: 0.338; 95%-CI: 0.311-0.365), followed by limb defects (n=362; prop.: 0.303; 95%-CI: 0.277-0.330), sensorineural defects (n=187; prop.: 0.157; 95%-CI: 0.136-0.177), musculoskeletal disorders (n=64; prop.: 0.054; 95%-CI: 0.041-0.066), visual impairments (n=64; prop.: 0.054; 95%-CI: 0.041-0.066), ectodermal disorders (n=40; prop.: 0.034; 95%-CI: 0.023-0.044), orofacial anomalies (n=34; prop.: 0.028; 95%-CI: 0.019-0.038), blood disorders (n=11; prop.: 0.009; 95%-CI: 0.004-0.015), and metabolic defects (n=8; prop.: 0.007; 95%-CI: 0.002-0.011).

**Table-II:** Major and minor categories of CA observed in study cohort.

Malformation (major/minor)	No. of cases	Proportion	95% CI	OMIM	ICD-10
***Neurological disorders***	403	0.338	0.311-0.365		
Intellectual disability (types)*	242	0.203	0.180-0.226	249500	F79
Epilepsy	30	0.025	0.016-0.034	604364	G40
Down syndrome	17	0.014	0.008-0.021	190685	Q90
Hydrocephaly	10	0.008	0.003-0.014	236600	G91.9
Microcephaly	9	0.008	0.003-0.012	608716	Q02
Autism	3	0.003	0.000-0.005	209850	F84.0
Macrocephaly	2	0.002	-0.001-0.004		Q75.3
Cerebral palsy	74	0.062	0.048-0.076	612900	G80.9
Spina bifida	9	0.008	0.003-0.012	182940	Q76.0
Meningocele	2	0.002	-0.001-0.004		Q05
Encephalocele	1	0.001	-0.001-0.002		Q01.9
Huntington chorea	1	0.001	-0.001-0.002	143100	G10
Cerebral ectopia	1	0.001	-0.001-0.002		Q04.8
Hyperkalemic periodic paralysis	1	0.001	-0.001-0.002	170500	G72.3
Multiple sclerosis	1	0.001	-0.001-0.002	126200	G35
***Limb defects***	362	0.303	0.277-0.330		
Polydactyly (types)*	205	0.172	0.150-0.193		Q69.0
Talipes (types)*	44	0.037	0.026-0.048	119800	Q66.6
Syndactyly (types)*	42	0.035	0.025-0.046	610713	Q70.9
Brachydactyly	18	0.015	0.008-0.022	113300	Q68.1
Club thumb	8	0.007	0.002-0.011		R68.3
Amputations	14	0.012	0.006-0.018		Y83.5
Constriction ring syndrome	5	0.004	0.001-0.008	217100	Q79.8
Radial hemimelia	2	0.002	-0.001-0.004		Q71.4
Symbrachydactyly	1	0.001	-0.001-0.002	113000	
Camptodactyly	12	0.010	0.004-0.016	114200	Q68.1
Clinodactyly	4	0.003	0.000-0.007		
Leg length discrepancy	4	0.003	0.000-0.007		Q72
Overriding toe	2	0.002	-0.001-0.004		
Arachnodactyly/Marfan syndrome	1	0.001	-0.001-0.002	154700	Q87.4
***Sensorineural defects***	187	0.157	0.136-0.177		
Deaf-mute (types)*	171	0.143	0.123-0.163	304500	H90
Stuttering	10	0.008	0.003-0.014	614668	F98.5
Microtia	6	0.005	0.001-0.009	600674	Q17.2
***Musculoskeletal disorders***	64	0.054	0.041-0.066		
Muscular dystrophy	21	0.018	0.010-0.025	310200	G71.0
Muscular atrophy	3	0.003	0.000-0.005	137370	Q74.1
Facio-scapulo-humoral muscular dystrophy	2	0.002	-0.001-0.004	158900	G71.0
Spinal muscular atrophy	1	0.001	-0.001-0.002	253300	G12.9
Dwarfism	17	0.014	0.008-0.021		
Scoliosis	1	0.001	-0.001-0.002	181800	M41.9
Kyphoscoliosis	7	0.006	0.002-0.010	610170	M41
Osteogenesis imperfecta (type-I)	2	0.002	-0.001-0.004	166200	Q78.0
Arthrogryposis (type-I)	2	0.002	-0.001-0.004	108120	Q68.8
Multiple pterygium syndrome	1	0.001	-0.001-0.002	265000	
DuPan syndrome	1	0.001	-0.001-0.002		
Exostosis	1	0.001	-0.001-0.002	133700	Q78.6
Friedreich’s ataxia	1	0.001	-0.001-0.002	601992	G11.1
Hip dislocation, gait problem	1	0.001	-0.001-0.002	142700	Q62.5
Pelvic girdle hypoplasia	1	0.001	-0.001-0.002	602484	Q74.2
Pfeiffer syndrome	1	0.001	-0.001-0.002	101600	B27
Shoulder dysplasia	1	0.001	-0.001-0.002	169550	
***Visual impairments***	64	0.054	0.041-0.066		
Blindness (unspecified)	32	0.027	0.018-0.036	216900	H54.9
Retinitis pigmentosa	4	0.003	0.000-0.007	268000	H35.5
Colour blindness	2	0.002	-0.001-0.004	303800	H53.5
Glaucoma	2	0.002	-0.001-0.004	231300	H40
Night blindness	1	0.001	-0.001-0.002	310500	H53.6
Squint eye/strabismus	13	0.011	0.005-0.017	185100	H50.9
Anophthalmia	6	0.005	0.001-0.009		Q11
Nystagmus	2	0.002	-0.001-0.004	310700	H55
Microphthalmia	1	0.001	-0.001-0.002	611040	Q11.3
Ptosis	1	0.001	-0.001-0.002	300245	Q10.0
***Ectodermal disorders***	40	0.034	0.023-0.044		
Psoriasis	10	0.008	0.003-0.014	605606	L40
Albinism	8	0.007	0.002-0.011	203100	E70.3
Ichthyosis	6	0.005	0.001-0.009	242300	Q80.2
Ectodermal dysplasia	6	0.005	0.001-0.009	305100	Q82.4
Ectodermal dysplasia-syndactyly syndrome	1	0.001	-0.001-0.002	613573	Q82.8
Nail dystrophy	5	0.004	0.001-0.008	161050	L60.3
Alopecia universalis	2	0.002	-0.001-0.004	203655	L63.1
Hermansky-Pudlak Syndrome	1	0.001	-0.001-0.002	203300	E70.3
Woolly hair	1	0.001	-0.001-0.002	278150	Q84.1
***Orofacial anomalies***	34	0.028	0.019-0.038		
Cleft lip and palate	18	0.015	0.008-0.022	119530	Q37.0
Cleft palate	7	0.006	0.002-0.010	119540	Q35
Cleft lip	7	0.006	0.002-0.010	119530	Q36
Ankyloglossia	2	0.002	-0.001-0.004	106280	Q38.1
***Blood disorders***	11	0.009	0.004-0.015		
Thalassemia	6	0.005	0.001-0.009	613985	D56
Hemophilia	5	0.004	0.001-0.008	306700	D66
Metabolic disorders	8	0.007	0.002-0.011		
Mucopolysaccharidosis	2	0.002	-0.001-0.004	309900	E76
Hypothyroidism	1	0.001	-0.001-0.002	218700	E03.1
Diabetes mellitus type-1	5	0.004	0.001-0.008	222100	E10
***Others***	20	0.017	0.009-0.024		
Bardet-Biedl syndrome	6	0.005	0.001-0.009	209900	Q87.8
Imperforate anus	3	0.003	0.000-0.005		Q42.3
Cardiac septal defect	6	0.005	0.001-0.009		Q21.9
Inflammatory bowel disease	1	0.001	-0.001-0.002	266600	
Lymphoedema	1	0.001	-0.001-0.002	153100	Q82.0
Diffuse mesangial sclerosis	1	0.001	-0.001-0.002	249660	N05
Polycystic kidney disease	1	0.001	-0.001-0.002	173900	Q61.3
Progeria	1	0.001	-0.001-0.002	176670	E34.8

The neurological disorders were further resolved into 15 subcategories, and among those, intellectual disability was prominent (n=242; 60%), followed by cerebral palsy (n=74), epilepsy (n=30) and Down syndrome (n=17). Among the limb defects, polydactyly was most prevalent (n=205; 57%), followed by talipes (n=44), syndactyly (n=42), brachydactyly (n=18), amputations (n=14), and camptodactyly (n=12) (Table-II). In sensorineural defects, the majority of the cases were presented with deaf-mute (n=171). In the musculoskeletal disorders, muscular dystrophy and dwarfism were prominent (n=21 and 17, respectively). In visual impairments, blindness and strabismus were common (n=32 and 13, respectively) (Table-II).

### Familial/sporadic nature and isolated/syndromic presentation:

The CA with familial occurrence were 381 (32%) compared to 812 (68%) cases with sporadic appearance (Table-III). Metabolic disorders were more often familial in nature (87%), while orofacial defects, limb defects, neurological disorders, and ectodermal disorders had a sporadic occurrence in most of the cases (79%, 74%, 73%, and 62%, respectively; differences were statistically significant).

**Table-III:** Major categories of CA with respect to gender, familial/sporadic nature, isolated/sporadic presentations.

Anomaly	Total, %	Gender		Familial/sporadic nature*		Isolated/syndromic*	

		Male	Female	Sporadic	Familial	Isolated	Syndromic
Neurological disorders	403 (34)	232 (58)	171 (42)	296 (73)	107 (27)	275 (68)	128 (32)
Limb defects	362 (30)	201 (56)	161 (44)	267 (74)	95 (26)	333 (92)	29 (8)
Sensorineural defects	187 (16)	130 (70)	57 (30)	112 (60)	75 (40)	175 (94)	12 (6)
Musculoskeletal disorders	64 (5)	34 (53)	30 (47)	37 (58)	27 (42)	45 (70)	19 (30)
Visual impairments	64 (5)	35 (55)	29 (45)	31 (48)	33 (52)	54 (84)	10 (16)
Ectodermal disorders	40 (3)	20 (50)	20 (50)	25 (62)	15 (38)	37 (92)	3 (8)
Orofacial anomalies	34 (3)	17 (50)	17 (50)	27 (79)	7 (21)	33 (97)	1 (3)
Blood disorders	11 (1)	7 (64)	4 (36)	5 (45)	6 (55)	10 (91)	1 (9)
Metabolic defects	8 (1)	5 (63)	3 (38)	1 (13)	7 (87)	6 (75)	2 (25)
Others	20 (2)	10 (50)	10 (50)	11 (55)	9 (45)	13 (65)	7 (35)
Total	1193 (100)	691 (58)	502 (42)	812 (68)	381 (32)	981 (82)	212 (18)

* Differences in the distribution were statistically significant.

The majority of the CA had an isolated presentation (n=981; 82%), while only 212 (18%) had a syndromic appearance (Table-III). Orofacial anomalies, sensorineural defects, ectodermal disorders, and limb defects were mostly isolated in nature (97%, 94%, 92% and 92%, respectively), whereas syndromic presentation was conspicuous among the metabolic defects, neurological disorders, and musculoskeletal disorders (25%, 32%, and 30%, respectively; differences were statistically significant).

### Familial cases and parental consanguinity:

The pedigree structures of the familial cases (n=381) were analyzed to score the disease segregating generations and affected family members. The majority of the CA involved a single generation or two generations (60% and 30%, respectively) (data not shown). Familial limb defects were an exception that appeared in two generations more often (43%). Further, the majority of the familial cases were presented with 2 or 3-4 affected individual per family (n=184, 48% and n=137, 36%, respectively). The total number of affected individuals in all familial cases was 1259 (721 males and 538 females, respectively).

In the overall cohort, 757 (63%) of the individual had parental consanguinity. There were marked differences in the distribution of consanguinity among the major categories of CA (p<0.0001). Parental consanguinity was also observed to be significantly higher among the familial cases compared to the sporadic anomalies (70% vs 60%; p>0.0001). However, the differences in the distribution of parental consanguinity between the male and female index cases, and isolated and sporadic cases were statistically not significant (p=0.43 and p=0.31, respectively) (data not shown).

### Syndromic anomalies:

Among the syndromic malformations, the six most common associations were deaf-mute, musculoskeletal disorders, limb defects, polydactyly, cerebral palsy, and squint eye (Table-IV).

**Table-IV:** Syndromic cases with combination of associated anomalies*

	Major category						

Associated anomaly	Neurological disorders	Limb defects	Musculoskeletal disorders	Sensorineural defects	Visual impairments	Others	Total
Deaf-mute	23	0	3	0	1	0	27
Musculoskeletal disorders**	21	0	0	2	0	0	23
Limb defects***	8	7	2	2	1	1	21
Polydactyly	5	1	2	2	1	4	15
Cerebral palsy	13	0	0	0	0	0	13
Squint eye	5	5	1	1	0	1	13
Ectodermal disorders	7	1	0	2	2	0	12
Stuttering	11	0	1	0	0	0	12
Epilepsy	11	0	0	0	0	0	11
Dwarfism	8	0	1	0	0	1	10
Visual anomalies****	6	1	2	0	0	0	9
Gait problem	3	1	2	1	2	0	9
Syndactyly	1	4	1	0	0	0	6
Others	9	4	1	2	0	0	16
Sum	131	24	16	12	7	7	197

* only the associations with n≥7 are shown;

** other than dwarfism and gait problem;

*** other than polydactyly and syndactyly;

**** other than squint eye.

## DISCUSSION

This study reports 1193 individual /families with CA ascertained from AJK region of Pakistan. There was a broad spectrum of anomalies involving various organ-systems, a pattern distinct from other hospital-based registries. Collectively, there were at least 85 distinct entities. Out of 226 congenitally malformed neonates Hussain et al.^13^ observed 47 different CA among the neonates in a teaching hospital in Kharian, Pakistan. In a cohort of 246 independent patients recruited from Kurram Tribal Agency, Pakistan, Zahra et al. witnessed that there were nine major and 49 minor categories of CA.^9^

In the present study, neurological disorders accounted for 34% of the total cases and there were 15 different subcategories. Among these, intellectual disability (ID) was most common (n=242). The highest representation of anomalies associated with central nervous system (CNS) was also reported by other researchers.^13^,^14^,^6^ Daliri et al.^15^ conducted meta-analyses on literature available for Iranian populations and revealed that musculoskeletal disorders and urogenital anomalies had the highest prevalence. This wide variability in the prevalence of CA in different populations may be attributed to the differences in the genetic backgrounds of populations, methods of ascertainments, risk factors, and categorization schemes.

Limb defects emerged as the second most common type of CA in this cohort. Certain limb defects of minor nature, for instance, partial cutaneous syndactyly, brachydactyly, clinodactyly, and camptodactyly, may skip the attention of the doctor. Further, owing to their non-lethal characteristics, the minor limb defects are usually ignored and remain under-reported.^16^ In this study, there was the highest representation of polydactyly (n=205; 57%). Among polydactyly types, there was nearly equal representation of preaxial and postaxial types while mesoaxial polydactyly was rare (data not shown). The high prevalence of polydactyly among the limb defects is concordant with other studies.^8^,^16^,^17^ Ullah et al. in a population-based study carried out in Chitral district of Pakistan, demonstrated that isolated polydactyly was the most common type of limb defect, followed by syndactyly and amputations.^16^ Clubfoot and polydactyly were the most frequent anomalies in cohorts reported from Iran and Pakistan.^18^-^20^ Limb defects mostly have sporadic and isolated nature which may be indicative of nongenetic and environmental etiology.^21^

In our cohort, the number of sporadic cases was higher than the familial cases which is concordant with the findings of Zahra et al.^9^ and Ullah et al.^16^ in other populations of Pakistan. A substantial number of sporadic cases may be due to *de novo* mutations caused by environmental or unknown factors. A number of environmental pollutants and heavy metals have an association with congenital anomalies.^21^

### Limitations:

First, this study does not provide the true prevalence and incidence rates of CA in the study population. Second, it does not provide any clue to which extent the CA are caused by the environmental factors, particularly the sporadic cases. This study, however, illustrates the pattern and phenotypic manifestations of CA prevalent in the AJK. Further, the familial cases may be followed up for the elucidation of molecular basis of these anomalies.

## CONCLUSIONS

Majority of the anomalies observed in this cohort are of severe nature rendering life-long disability and poor quality of life, and require early detection, intervention and management. These data are vital for policymakers in resource allocation, setting priorities, planning and implementing health services and genetic counseling services. In a wider perspective, the country-wide registry is essential to establish a baseline prevalence of CAs.

## References

[1] WHO/Congenitalanomalies [Internet] WHO.

[2] Corsello G, Giuffrè M, Buonocore G (2012). Congenital Malformations and Syndromes:Early Diagnosis and Prognosis. Neonatology. A Practical Approach to Neonatal Diseases.

[3] Singh K, Krishnamurthy K, Greaves C, Kandamaran L, Nielsen AL, Kumar A (2014). Major congenital malformations in Barbados:the prevalence, the pattern, and the resulting morbidity and mortality. ISRN Obstet Gynecol.

[4] Sokal R, Tata LJ, Fleming KM (2014). Sex prevalence of major congenital anomalies in the United Kingdom:a national population-based study and international comparison meta-analysis. Birth Def Res.

[5] Fida NM, Al-Aama J, Nichols W, Nichols W, Alqahtani M (2007). A prospective study of congenital malformations among liveborn neonates at a University Hospital in Western Saudi Arabia. Saudi Med J.

[6] Bibi A, Naqvi SF, Syed A, Zainab S, Sohail K, Malik S (2022). Burden of Congenital and Hereditary Anomalies in Hazara Population of Khyber Pakhtunkhwa, Pakistan. Pak J Med Sci.

[7] Nawaz A, Zaman M, Malik S (2021). Consanguinity, inbreeding coefficient, fertility and birth-outcome in population of Okara district. Pak J Med Sci.

[8] Lal K, Malik S (2015). Epidemiological study of congenital limb defects in individuals or families from the interior Sindh region of Pakistan. Asian Biomed.

[9] Zahra Q, Shuaib M, Malik S (2016). Epidemiology of congenital anomalies in the Kurram Tribal Agency, northwest Pakistan. Asian Biomed.

[10] ABS. AJ&K Bureau of Statistics Azad Jammu and Kashmir Statistical Year Book Azad Government of the State of Jammu and Kashmir.

[11] Jabeen N, Malik S (2014). Consanguinity and its sociodemographic differentials in Bhimber District, Azad Jammu and Kashmir, Pakistan. J Health Pop Nutrit.

[12] World Medical Association. World Medical Association Declaration of Helsinki:ethical principles for medical research involving human subjects (2013). JAMA.

[13] Hussain S, Asghar I, Sabir MU, Chattha MN, Tarar SH, Mushtaq R (2014). Prevalence and pattern of congenital malformations among neonates in the neonatal unit of a teaching hospital. J Pak Med Assoc.

[14] Khan A, Zuhaid M, Fayaz M, Ali F, Khan A, Ullah R (2015). Frequency of congenital anomalies in newborns and its relation to maternal health in a Tertiary Care Hospital in Peshawar, Pakistan. Int J Med Students.

[15] Daliri S, Sayehmiri K, Asadollahi Kh, Rezaei N, Saroukhani D, Karimi A (2018). Prevalence of congenital anomalies in Iran:a systematic review and meta-analysis. Iran J Neonatol.

[16] Ullah S, Dasti JI, Malik S (2015). Descriptive epidemiology of hereditary musculoskeletal and limb defects in the isolated population of Chitral, North West Pakistan. Pak J Med Sci.

[17] Vasluian E, Van der Sluis CK, Van Essen AJ, Bergman JE, Dijkstra PU, Reinders-Messelink HA (2013). Birth prevalence for congenital limb defects in the northern Netherlands:a 30-year population-based study. BMC Musculoskelet Disord.

[18] Golalipour MJ, Ahmadpour-Kacho M, Vakili MA (2005). Congenital malformations at a referral hospital in Gorgan, Islamic Republic of Iran. East Mediter Health J.

[19] Naeem M, Ahmad B, Malik S (2022). Burden of congenital and hereditary anomalies in the war-affected territory at Pakistan-Afghanistan border. Asian Biomed.

[20] Bhatti NA, Mumtaz S, Malik S (2019). Epidemiological study of congenital and hereditary anomalies in Sialkot District of Pakistan revealed a high incidence of limb and neurological disorders. Asian Biomed.

[21] Vrijheid M, Martinez D, Manzanares S, Dadvand P, Schembari A, Rankin J (2011). Ambient air pollution and risk of congenital anomalies:a systematic review and meta-analysis. Environ Health Perspect.

